# Cer-ConvN3Unet: an end-to-end multi-parametric MRI-based pipeline for automated detection and segmentation of cervical cancer

**DOI:** 10.1186/s41747-025-00557-2

**Published:** 2025-02-18

**Authors:** Shao-Jun Xia, Bo Zhao, Yingming Li, Xiangxing Kong, Zhi-Nan Wang, Qingmo Yang, Jia-Qi Wu, Haijiao Li, Kun Cao, Hai-Tao Zhu, Xiao-Ting Li, Xiao-Yan Zhang, Ying-Shi Sun

**Affiliations:** 1 Key Laboratory of Carcinogenesis and Translational Research (Ministry of Education/Beijing), Department of Radiology, Peking University Cancer Hospital & Institute, Beijing, China; 2 Department of Radiology, Peking University People’s Hospital, Beijing, China; 3 Key Laboratory of Carcinogenesis and Translational Research (Ministry of Education/Beijing), Key Laboratory for Research and Evaluation of Radiopharmaceuticals (National Medical Products Administration), Department of Nuclear Medicine, Peking University Cancer Hospital & Institute, Beijing, China; 4 Peking University School and Hospital of Stomatology, Beijing, China

**Keywords:** Artificial intelligence, Automated detection and segmentation, Deep learning, Magnetic resonance imaging, Uterine cervical neoplasms

## Abstract

**Background:**

We established and validated an innovative two-phase pipeline for automated detection and segmentation on multi-parametric cervical cancer magnetic resonance imaging (MRI) and investigated the clinical efficacy.

**Methods:**

The retrospective multicenter study included 125 cervical cancer patients enrolled in two hospitals for 14,547 two-dimensional images. All the patients underwent pelvic MRI examinations consisting of diffusion-weighted imaging (DWI), T2-weighted imaging (T2WI), and contrast-enhanced T1-weighted imaging (CE-T1WI). The deep learning framework involved a multiparametric detection module utilizing ConvNeXt blocks and a subsequent segmentation module utilizing 3-channel DoubleU-Nets. The pipeline was trained and tested (80:20 ratio) on 3,077 DWI, 2,990 T2WI, and 8,480 CE-T1WI slices.

**Results:**

In terms of reference standards from gynecologic radiologists, the first automated detection module achieved overall results of 93% accuracy (95% confidence interval 92–94%), 93% precision (92–94%), 93% recall (92–94%), 0.90 κ (0.89–0.91), and 0.93 F1-score (0.92–0.94). The second-stage segmentation exhibited Dice similarity coefficients and Jaccard values of 83% (81–85%) and 71% (69–74%) for DWI, 79% (75–82%), and 65% (61–69%) for T2WI, 74% (71–76%) and 59% (56–62%) for CE-T1WI.

**Conclusion:**

Independent experiments demonstrated that the pipeline could get high recognition and segmentation accuracy without human intervention, thus effectively reducing the delineation burden for radiologists and gynecologists.

**Relevance statement:**

The proposed pipeline is potentially an alternative tool in imaging reading and processing cervical cancer. Meanwhile, this can serve as the basis for subsequent work related to tumor lesions. The pipeline contributes to saving the working time of radiologists and gynecologists.

**Key Points:**

- An AI-assisted multiparametric MRI-based pipeline can effectively support radiologists in cervical cancer evaluation.
- The proposed pipeline shows high recognition and segmentation performance without manual intervention.
- The proposed pipeline may become a promising auxiliary tool in gynecological imaging.

**Graphical Abstract:**

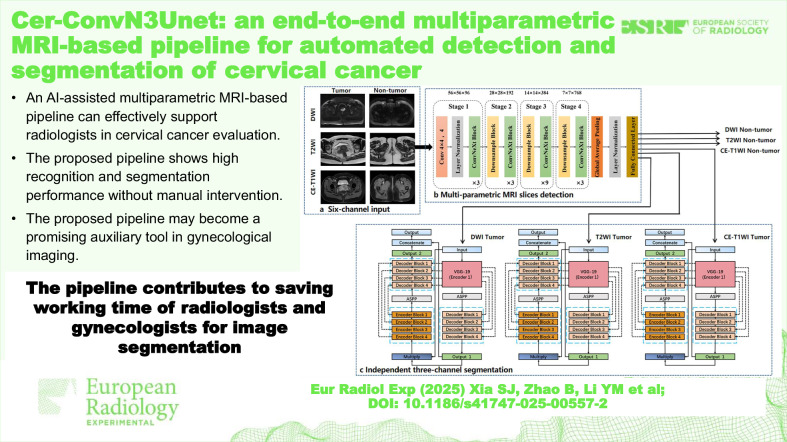

## Background

Cervical cancer is the fourth leading cause of cancer-related deaths in women [1–3]. Magnetic resonance imaging (MRI), including T2-weighted imaging (T2WI), contrast-enhanced T1-weighted imaging (CE-T1WI), and diffusion-weighted imaging (DWI), is the standard imaging tool for preoperative local staging of cervical cancer in clinical practice [4]. These imaging methods enable the assessment of the local extent of the tumor and its maximum diameter. Moreover, with the implantation of radiomics, radiologists cannot quantify intratumoral heterogeneity by extracting mineable high-dimensional imaging features from tumors [5, 6]. Accurate extraction of lesions contributes to expediting the procedure of radiotherapy [7]. However, selecting the region of interest (ROI) for a tumor using radiomics analysis or clinical radiation practice is time-consuming because the number of tumor layers is often less than the non-tumor layers [8]. On the other hand, manual delineation brings an increased burden for radiologists due to the random tumor distribution adhering to the cervix uteri [9, 10]. Thus, developing a highly efficient lesion extraction method for multi-parametric MRI images may improve research processes and clinical treatment of cervical cancer.

Several preliminary studies have examined MRI methods before treatment to predict lymph node metastasis [11], evaluate survival [12], and assess treatment response in cervical cancer [13]. Other studies demonstrate that the radiomics signature from multi-parametric MRI images performs better in the prediction of lymphovascular space invasion than when single-sequence MRI is used [14, 15]. Multi-parametric MRI incorporates multiple sequence acquisitions that highlight anatomical and functional information in tumors, which may reduce the risk of bias in the radiomics features obtained from a single sequence [11, 12]. Recent advancements in deep learning (DL), including convolutional neural networks (CNNs), have been proven to have excellent performance in the general use of multiple detection and segmentation tasks in medical imaging [16–20], particularly for brain or cardiac diseases. Nevertheless, research on cervical cancer detection and segmentation has predominantly focused on single-sequence MRI or computed tomography (CT) images [8, 21–27], with relatively few systematic investigations of the segmentation performance across different sequences. Some studies have explored the analysis of multi-parametric MRI of cervical cancer but are primarily constrained by either limited sequences [28, 29] or non-DL algorithms [30].

In this study, we hypothesized that (i) advanced deep learning architectures can be leveraged into an intelligent integrated pipeline for (a) detecting and (b) segmenting cervical cancer lesions in multi-parametric MRI images with high accuracy; (ii) the developed DL-based pipeline can reduce the time required for tumor delineation and improve the efficiency for gynecologic radiologists (GRs); (iii) the trained pipeline in one center is supposed to have stable generalization performance in unseen patients collected from another center, thus potentially assist research processes and clinical treatment of cervical cancer. Therefore, this study aimed to construct an innovative two-phase pipeline for automated detection and segmentation on multi-parametric cervical cancer MRI images and investigate the effectiveness of GRs. The novelty of the study can be summarized into four items: (1) herein, we proposed an innovative end-to-end multi-parametric pipeline for cervical cancer detection and segmentation with no human intervention/interaction required by providing accurate detection and segmentation results in multi-parametric MRI tumor slices; (2) this is the first study which attempted to apply the pre-trained ConvNeXt [31] model in the multi-sequence domain of cervical cancer; the two-stage design effectively combines the advantages of ConvNeXt in classification and DoubleU-Net in segmentation; (3) the DL-based pipeline demonstrates comprehensive performances in terms of auto-delineation accuracy, time savings, and generalization ability on external validation; (4) this study systematically expands the understanding of the segmentation performance across different imaging sequences, shedding light on downstream multi-sequence approaches for cervical cancer analysis, *e.g.*, DL-based radiomics and DL-assisted radiotherapy. These data may have a far-reaching influence on subsequent lesion-related research processes and clinical treatment, such as tumor measurement, radiomics analysis, radiotherapy treatment planning, and surgical preparation.

## Methods

### Patients

This retrospective study did not include any interventions on patient examination sequences or treatments. The ethics committee waived the requirement for informed consent.

A total of 125 women with cervical cancer treated in the two disparate hospitals (Center 1 and Center 2) from February 2013 to November 2016 and from January 2022 to December 2022 were analyzed. Patients underwent three sequences of DWI, T2WI, and CE-T1WI scans. The inclusion criteria were the following: (1) histologically confirmed cervical squamous cell carcinoma, adenocarcinoma, and neuroendocrine; (2) patients who underwent routine contrast-enhanced pelvic MRI, including axial DWI, axial T2WI, and axial CE-T1WI sequence; (3) acceptable quality of MR images; (4) no history of treatment before MRI examination. In this multi-center study, Center 1 was the primary center, conducting most of the experiments; Center 2 was the secondary center, mainly providing cross-center validation.

### MRI protocol

All patients underwent routine contrast-enhanced pelvic MRI before treatment. Each patient was exposed to 3D scanning and 375 3D MRI images were acquired at this stage. Afterward, the acquired 3D images were converted into 2D slices to fit the subsequent pipeline development. MRI acquisitions were performed on five scanning devices (Center 1: United Imaging 3.0-T—uMR790, SIEMENS 1.5-T—Aera, GE 3.0-T—Discovery750; Center 2: United Imaging 1.5-T—uMR588, GE 3.0-T—Discovery750). Axial delayed-phase contrast-enhanced T1-weighted images were collected 2–4 min after intravenous administration of contrast agent (gadodiamide, 0.1 mmol/kg; Omniscan; GE Healthcare, Co. Cork, Ireland) injected at a rate of 2.0 mL/s, followed by a 20-mL saline flush. Patients with no contraindications received an intramuscular injection of 10 mg raceanisodamine hydrochloride before image acquisition to reduce bowel motion artifacts. Detailed information on the three sequences is listed in Table 1.

**Table 1:** MRI parameters of scanning sequences with DWI, T2WI, and CE-T1WI

	Scanner	Patient	Sequence	Pulse sequence	TR (ms)	TE (ms)	Matrix	FOV	ST (mm)	SG (mm)	FA
Center 1	United Imaging 3.0-T (uMR790)	55	DWI	EPI	2,000	60	128 × 110	350 × 329	5	6	90
			T2WI	FSE	3,057	147.52	336 × 336	276 × 260	5	6	130
			CE-T1WI	GRE	3.98	1.82	272 × 244	276 × 260	2	2	12
	SIEMENS 1.5-T (Aera)	20	DWI	EPI	2,000	60	128 × 110	350 × 329	5	6	90
			T2WI	SE	2,000	98	384 × 288	276 × 260	5	6	126
			CE-T1WI	GRE	9.51	2.39	320 × 195	320 × 282	3	2	10
	GE 3.0-T (Discovery 750)	25	DWI	EPI	3,048	73.50	130 × 100	364 × 340	5	6	90
			T2WI	SE	7,142	110.60	320 × 288	278 × 260	5	6	145
			CE-T1WI	GRE	3.84	1.67	260 × 256	364 × 340	4	2	12
Center 2	United Imaging 1.5-T (uMR 588)	9	DWI	EPI	2,400	60	160 × 160	340 × 340	6	7.50	90
			T2WI	FSE	4,100	85	384 × 224	340 × 340	6	7.50	111
			CE-T1WI	GRE	3.92	1.76	288 × 224	380 × 340	2	0	12
	GE 3.0-T (Discovery750)	16	DWI	EPI	4,500	87.40	208 × 256	260 × 320	5	6.50	90
			T2WI	FSE	5,000	85	348 × 504	260 × 320	5	6.50	120
			CE-T1WI	GRE	4.05	1.66	312 × 480	260 × 320	3	0	0

*CE-T1WI* Contrast-enhanced T1-weighted imaging, *DWI* Diffusion-weighted imaging, *EPI* Echo-planar imaging, *FOV* Field of view, *GRE* Gradient echo, *MRI* Magnetic resonance imaging, *SE* Spin echo, *SG* Section gap, *ST* Section thickness, *T2WI* T2-weighted imaging, *TE* Echo time, *TR* Repetition time

### ROIs delineation and image annotation

**Fig. 1:**
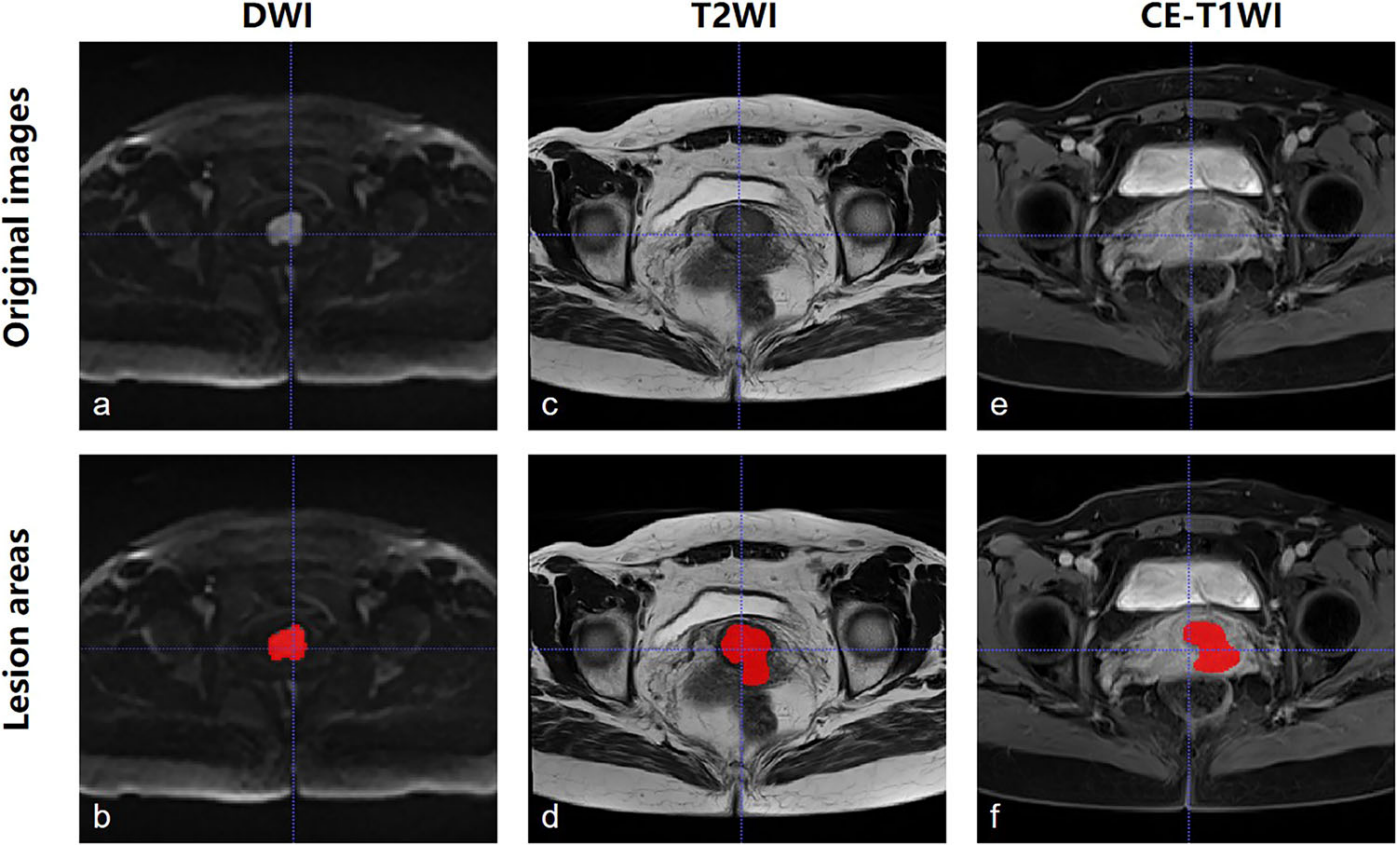
Example images from a 49-year-old woman with cervical cancer on DWI, T2WI, and CE-T1WI, with corresponding radiologist-delineated lesion regions. **a**, **b** DWI image and ROI image, *b* = 1,000 s/mm^2^. **c**, **d** T2WI image and ROI image. **e**, **f** CE-T1WI image and ROI image. CE-T1WI, Contrast-enhanced T1-weighted imaging; DWI, Diffusion-weighted imaging; ROI, Region of interest; T2WI, T2-weighted imaging

DWI, T2WI, and CE-T1WI images in NIfTI format were obtained after scanning. ITK-SNAP software (version 3.8; www.itksnap.org) was used for manual contour delineation. The tumor areas on the axial DWI images, axial T2WI images, and axial CE-T1WI images were first outlined by a radiologist (with 7 years of gynecologic imaging experience referring to the sagittal T2WI and axial ADC images) and then reviewed and corrected by a senior attending radiologist (with more than 15 years of gynecologic imaging experience). The outlined ROIs were also transferred to NIfTI format by ITK-SNAP software, as shown in Fig. 1. Next, the 3D MRI images in section 2.2 were extracted into 2D slices layer by layer using Python 3.6 and the SimpleITK library. Slices with outlined ROIs were labeled as tumor images, and the others were labeled as non-tumor images. A total of six types of slices were encompassed, including DWI tumor, DWI non-tumor, T2WI tumor, T2WI non-tumor, CE-T1WI tumor, and CE-T1WI non-tumor slices.

### Overview of the pipeline

**Fig. 2:**
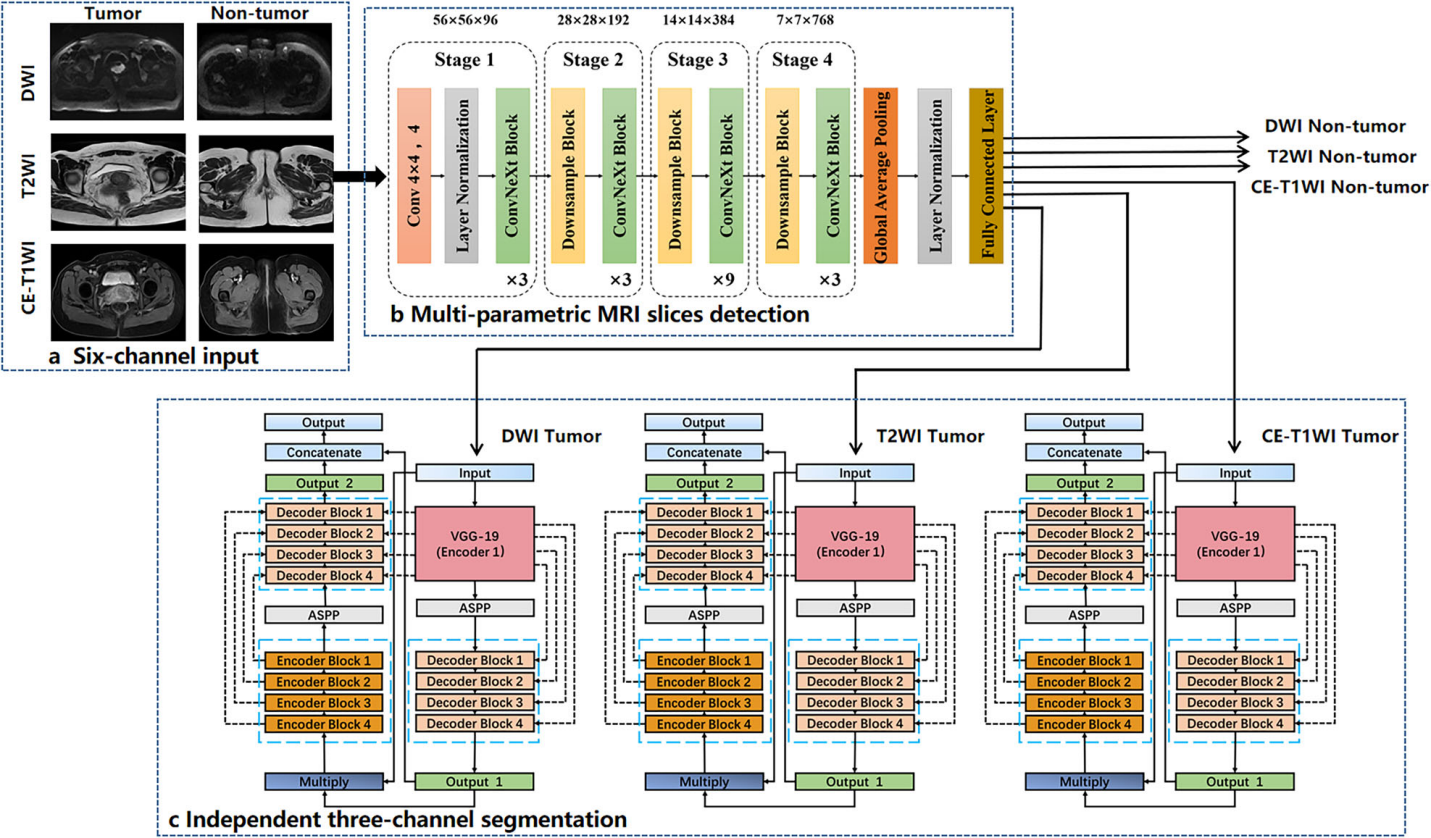
Schematic representation of the overall pipeline for automated detection and auto-segmentation of cervical cancer—Cer-ConvN3Unet. **a** Six-channel input module: DWI tumor slice, DWI non-tumor slice, T2WI tumor slice, T2WI non-tumor slice, CE-T1WI tumor slice, and CE-T1WI non-tumor slice. **b** Multi-parametric MRI slices detection module based on ConvNeXt structure. **c** Independent three-channel segmentation module based on DoubleU-Net architecture. ASPP, Atrous spatial pyramid pooling; CE-T1WI, Contrast-enhanced T1-weighted imaging; DWI, Diffusion-weighted imaging; MRI, Magnetic resonance imaging; T2WI, T2-weighted imaging

In real-world medical image interpretation scenarios, addressing the issue of a significant proportion of pure negative samples (MRI slices without tumors) is crucial for applying 2D networks to cervical cancer images [32, 33]. Hence, proactively distinguishing pure negative samples may enhance the effectiveness of models in addressing imbalanced positive and negative data during training. As illustrated in Fig. 2, we presented a new pipeline, *i.e.*, Cer-ConvN3Unet, for automated detection and segmentation of cervical cancer. The pipeline includes three key components: a six-channel input module, a multi-parametric MRI detection module, and an independent three-channel segmentation module.

All the 3D MRI images were split into 2D slices, resized to 224 × 224 pixels, and then stacked three times in depth to get the same input dimension of 224 × 224 × 3. A multi-parametric MRI slices detection module based on ConvNext [31] was constructed to distinguish the presence or absence of tumors on every DWI, T2WI, and CE-T1WI slice. The backbone of ConvNeXt is divided into four different stages; each stage is composed of a downsample block and a ConvNeXt block. To balance the lightness and recognition accuracy of the network, this study mainly adopted the configuration of ConvNeXt-T, with the lightweight block ratio of 3:3:9:3. The dimensions at each stage are 56 × 56 × 56, 28 × 28 × 192, 14 × 14 × 384, 7 × 7 × 768, respectively. Moreover, we conducted numerous comparative experiments on the dataset of Center 1, which were associated with diverse ConvNeXt variants (ConvNeXt-S, ConvNeXt-B, ConvNeXt-L, ConvNeXt-XL; block ratio: 3:3:27:3) on two scales of ImageNet datasets (ImageNet-1K, ImageNet-22K).

After discarding the slices identified as a tumor-free class, three MRI images predicting tumor regions were obtained and used as the input of an independent three-channel segmentation module. Performing registration in multiple sequences before segmentation is often impractical and cumbersome in clinical scenarios [34]. Thus, the overall module consisted of three parallel DoubleU-Net [35] networks, each dedicated to the fine-grained segmentation of different sequences of images. The first U-Net utilizes a pre-trained VGG-19 as an encoder, enabling the transfer of learned features from ImageNet to other tasks. To capture more semantic information, the network incorporates an additional U-Net network with atrous spatial pyramid pooling for capturing contextual information. Each DoubleU-Net contains two atrous spatial pyramid pooling modules. The output results of the first U-Net and the second U-Net are concatenated to get the final segmentation results. This study used the independent three-channel design to autonomously process tumorous images across three distinct sequences. This stratagem is employed to circumvent the attenuation of network performance induced by the amalgamation of disparate sequence information. Ultimately, the DL pipeline furnishes corresponding outcomes for the multi-parametric MRI images. The sample images, the codes for detection and segmentation, and the sample results are available at: https://github.com/Post-nCRT/Cer-ConvN3Unet.

### Performance metrics and statistical analysis

#### Detection

Accuracy was calculated as the proportion of correctly predicted samples in all samples, expressed as the ratio of true positives (TPs) and true negatives (TNs) to the sum of false positives (FPs), TPs, TNs, and false negatives (FNs).

1 $${{{\rm{Accuracy}}}}=\frac{{{\rm{TP}}}+{TN}}{{{\rm{TP}}}+{TN}+{FP}+{FN}}$$

Precision was calculated as the proportion correctly classified into the positive sample category, *i.e.*, the classification accuracy of the positive sample. From the perspective of prediction results, it describes how many positive examples predicted by the classifier are accurate.

2 $${{{\rm{Precision}}}}=\frac{{{\rm{TP}}}}{{{\rm{TP}}}+{FP}}$$

The recall was calculated as the proportion of TPs in all positive samples currently assigned to the positive sample category. In terms of real results, it describes how many real positive examples are recalled by the classifier.

3 $${{{\rm{Recall}}}}=\frac{{{\rm{TP}}}}{{{\rm{TP}}}+{FN}}$$

*κ* statistics was used for assessing inter-rater reliability, *i.e.*, the agreement between two observers when categorizing or classifying data. The κ value ranges from -1 to 1. ${{{{\boldsymbol{r}}}}}_{{{{\boldsymbol{i}}}}}$ and ${{{{\boldsymbol{c}}}}}_{{{{\boldsymbol{i}}}}}$ represent the sum of the *i*th row and the *i*th column of the confusion matrix, respectively; ${{{\boldsymbol{n}}}}$ denotes the sum of the classified samples.

4 $${{{\rm{\kappa }}}}=\frac{{p}_{o}-{p}_{e}}{1-{p}_{e}}$$

5 $${p}_{o}={{{\rm{Accuracy}}}}$$

6 $${p}_{e}=\frac{{\sum }_{i}{r}_{i}* {c}_{i}}{{n}^{2}}$$

F1-score was used as a comprehensive indicator of the classification problem. It is a weighted average of precision and recall, with values from 0 to 1.

7 $${F}_{1}\, {{{\rm{score}}}}=\frac{2* {{{\rm{Precision}}}}* {{{\rm{Recall}}}}}{{{{\rm{Precision}}}}\,+\,{{{\rm{Recall}}}}}$$

The area under the curve (AUC) of the receiver operating characteristic (ROC) analysis was used to quantify the overall performance of a classification or detection model.

#### Segmentation

Dice similarity coefficient (DSC) was used to measure the similarity between two data sets in the range of [0, 1]. ${{X}}{{\cap }}{{Y}}$ is the intersection between ground truth (*X*) and prediction (*Y*) while $\left|{{X}}\right|$ and $\left|{{Y}}\right|$ denote the number of elements of *X* and *Y*, respectively.

8 $${{{\rm{DSC}}}}=\frac{2\left|X\cap Y\right|}{\left|X\right|+\left|Y\right|}=\frac{2{{{\rm{TP}}}}}{{{\rm{FP}}}+2{TP}+{FN}}$$

The Jaccard index was used to compare the similarities between a finite set of samples. It is defined as the ratio between the size of the intersection of two sets and the size of the union.

9 $${{{{\rm{Jaccard}}}\; {\rm index}}}=\frac{\left|X\cap Y\right|}{\left|X\cup Y\right|}=\frac{{{{\rm{TP}}}}}{{{{\rm{{FP}}}+{TP}+{FN}}}}$$

Statistical analyses were conducted using IBM SPSS Statistic 27 software. A paired *t*-test and a Wilcoxon signed-rank test were utilized to evaluate the time differences between the pipeline and manual delineation.

### Experimental device, process, and parameter details

The network architecture was developed and trained on a workstation with three GeForce RTX 2080 GPUs (PyCharm 2020.3, Python 3.7, PyTorch 1.7.0, Linux system, Ubuntu 16.04 server). In the auto-detection stage, all the six-channel images were resized to 224 × 224 × 3, the training epochs were set to 200 (bath size = 8), and a cross-entropy loss was optimized by AdamW (learning rate = 5e - 4, weight decay = 5e - 2). In the segmentation stage, the input size was 256 × 256 × 3 with the same batch size of 8 for 200 epochs. Dice loss and Adam optimizer (learning rate = e - 2) were utilized to train the segmentors. Training curves of the multi-parametric MRI detection module and the independent three-channel segmentation module are depicted in Figs. S1 and S2.

## Results

### Clinical characteristics of multiparametric MRI dataset

A total of 125 cervical cancer patients were used to create the multi-parametric MRI dataset. Complete sequences of DWI, T2WI, and CE-T1WI were available for all patients. A total of 100 patients (Center 1) were divided into the training cohort (including 20% for the validation cohort, median age: 56.00 [50.75, 62.00] years) and the internal testing cohort (median age: 55.50 [50.00, 62.50] years) based on a ratio of 4:1. A total of 25 patients (median age: 60.00 [47.00, 64.00] years) from Center 2 were used as the external testing cohort. The main clinical characteristics referring to the International Federation of Gynecology and Obstetrics (2018) stage [36] are summarized in Table 2. Following the slicing operation in section 2.3, the 2D dataset eventually comprised 14,547 multi-parametric MRI images, with 3,077 DWI, 2,990 T2WI, and 8,480 CE-T1WI slices, respectively.

**Table 2:** Patient characteristics of the training, validation, and testing cohorts with 2D slices

Characteristics		Center 1		Center 2	
		Training and validation cohort (*n* = 80)	Internal testing cohort (*n* = 20)	External testing cohort (*n* = 25)	*p*-value
Age (median [IQR])		56.00 [50.75, 62.00]	55.50 [50.00, 62.50]	60.00 [47.00, 64.00]	0.916^*^
FIGO (2018) stage (%)	I	35 (43.75)	5 (25.00)	8 (32.00)	0.185^#^
	II	23 (28.75)	8 (40.00)	12 (48.00)	
	III	21 (26.25)	5 (25.00)	4 (16.00)	
	IV	1 (1.25)	2 (10.00)	1 (4.00)	
Histologic subtype (%)	Squamous cell carcinoma	73 (91.25)	17 (85.00)	22 (88.00)	0.651^#^
	Adenocarcinoma	6 (7.5)	2 (10.00)	3 (12.00)	
	Neuroendocrine	1 (1.25)	1 (5.00)	0 (0.00)	
Tumor slices	DWI	462	112	122	
	T2WI	450	111	121	
	CE-TIWI	1,378	294	215	
Non-tumor slices	DWI	1,557	402	422	
	T2WI	1,506	381	421	
	CE-TIWI	4,488	1,378	727	

*CE-T1WI* Contrast-enhanced T1-weighted imaging, *DWI* Diffusion-weighted imaging, *FIGO* International Federation of Gynecology and Obstetrics, *IQR* interquartile range, *T2WI* T2-weighted imaging

^*^ Mann–Whitney *U*-test

^#^ Pearson chi-square test

### Multiparametric MRI-based detection performance of the DL pipeline

**Fig. 3:**
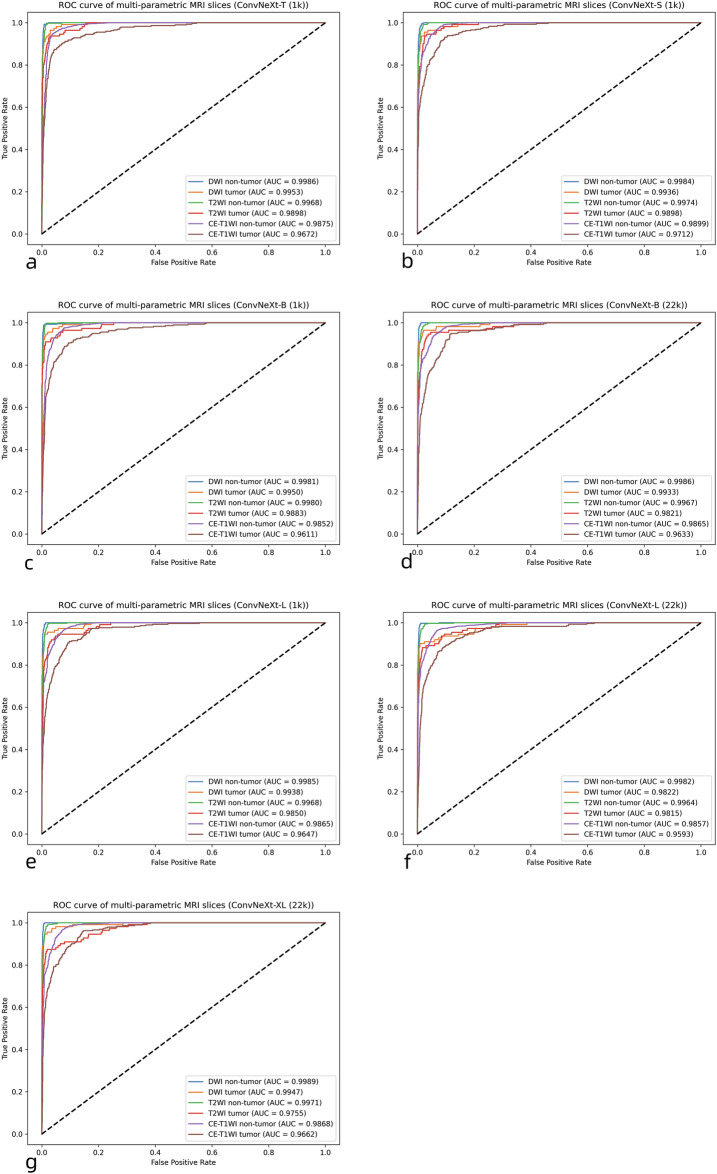
ROC curves and AUC values of the multi-parametric MRI slices detection module on the testing set, using different pre-trained ConvNeXt variants and ImageNet datasets (ImageNet-1K, ImageNet-22K). **a** ConvNeXt-T, ImageNet-1K. **b** ConvNeXt-S, ImageNet-1K. **c** ConvNeXt-B, ImageNet-1K. **d** ConvNeXt-B, ImageNet-22K. **e** ConvNeXt-L, ImageNet-1K. **f** ConvNeXt-L, ImageNet-22K. **g** ConvNeXt-XL, ImageNet-22K. AUC, Area under the curve; CE-T1WI, Contrast-enhanced T1-weighted imaging; DWI, Diffusion-weighted imaging; MRI, Magnetic resonance imaging; ROC, Receiver operating characteristics; T2WI, T2-weighted imaging

The multiparametric detection results and confidence intervals are shown in Table 3. The initial auto-detection module (ConvNeXt-T, pre-trained on ImageNet-1K) achieved the overall image-level results of 93% (92–94%), 93% (92–94%), 93% (92–94%), 0.90 (0.89–0.91), and 0.93 (0.92–0.94) for accuracy, precision, recall, κ, and F1-score at Center 1. Almost no error occurred when considering the ability to identify three different sequences. ROC curves and AUC values of the multi-parametric MRI slices detection module are visually depicted in Fig. 3a. For each type of slice, the module achieved high AUCs from 0.96 to 0.99. These results demonstrate that the multi-parametric MRI detection module can distinguish tumor presence at the slice level with high precision (Hypothesis i-a), excluding tumor-free slices and enabling sequential analysis for the independent three-channel segmentation module.

**Table 3:** The test performance for detecting multi-parametric MRI images (Center 1)

Pre-trained model		Precision	Recall	F1-score	κ	Accuracy
ConvNeXt-T (1k, separated slice types)	DWI non-tumor	96% (95–96%)	99% (98–99%)	0.97 (0.96–0.98)		
	DWI tumor	94% (93–95%)	84% (83–85%)	0.89 (0.88–0.90)		
	T2WI non-tumor	94% (93–95%)	96% (95–97%)	0.95 (0.94–0.96)		
	T2WI tumor	85% (83–86%)	80% (79–82%)	0.82 (0.81–0.84)		
	CE-T1WI non-tumor	95% (94–96%)	96% (95–96%)	0.95 (0.95–0.96)		
	CE-T1WI tumor	79% (78–81%)	78% (76–79%)	0.79 (0.77–0.80)		
ConvNeXt-T (1k)		93% (92–94%)	93% (92–94%)	0.93 (0.92–0.94)	0.90 (0.89–0.91)	93% (92–94%)
ConvNeXt-S (1k)		91% (90–93%)	91% (90–93%)	0.91 (0.90–0.92)	0.87 (0.86–0.89)	91% (90–93%)
ConvNeXt-B (1k)		92% (91–93%)	92% (91–93%)	0.92 (0.91–0.93)	0.88 (0.87–0.90)	92% (91–93%)
ConvNeXt-B (22k)		91% (90–92%)	91% (90–92%)	0.91 (0.90–0.92	0.87 (0.86–0.88)	91% (90–92%)
ConvNeXt-L (1k)		91% (90–93%)	92% (91–93%)	0.92 (0.90–0.93)	0.88 (0.86–0.89)	92% (91–93%)
ConvNeXt-L (22k)		91% (90–92%)	91% (90–92%)	0.91 (0.90–0.92)	0.87 (0.85–0.88)	91% (90–92%)
ConvNeXt-XL (22k)		92% (91–93%)	92% (91–93%)	0.92 (0.91–0.93)	0.88 (0.87–0.89)	92% (91–93%)

*CE-T1WI* Contrast-enhanced T1-weighted imaging, *DWI* Diffusion-weighted imaging, *MRI* Magnetic resonance imaging, *T2WI* T2-weighted imaging

The last six rows in Table 3 summarize the evaluation metrics for the other four ConvNeXt variants (ConvNeXt-S, ConvNeXt-B, ConvNeXt-L, and ConvNeXt-XL), and the corresponding AUC curves are provided in Fig. 3b–g. Compared to deeper network variants, using the lightest ConvNeXt-T model does not lead to a decline in performance. The consequences substantiate the superiority of the lightweight detection module in our pipeline design. From the perspective of the imaging sequence, the module performed best in recognizing DWI images (F1-score: 0.97, 0.89), while the results for T2WI images (F1-score: 0.95, 0.82) and CE-T1WI images (F1-score: 0.95, 0.79) were lower, which is also consistent with clinical experience in the diagnosis of cervical cancer, where DWI is considered the preferred sequence.

### Analysis of segmentation effects among different sequences based on DL pipeline

**Fig. 4:**
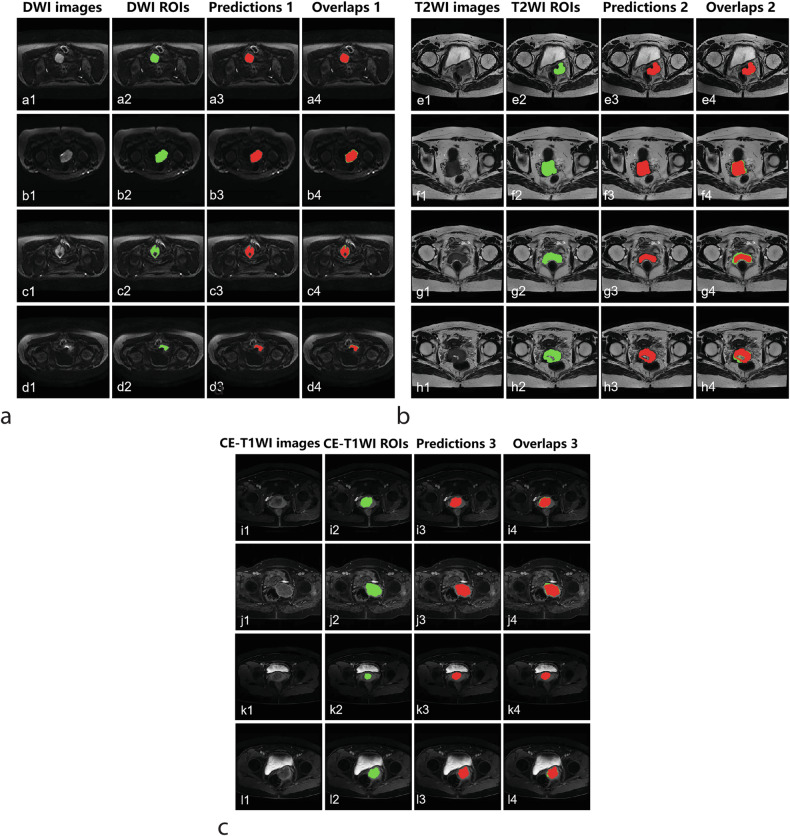
Four groups of segmentation samples for different sequences using the Cer-ConvN3Unet on the testing set. The green color shows the ground truth delineated by radiologists, while the red color shows the segmentation results. Meanwhile, the comparisons of the ground truth and the segmentation results are further illustrated by the overlaps (**a**) a1–d1: DWI images. a2–d2: DWI ROIs. a3–d3: Predictions 1. a4–d4: Overlaps 1. **b** e1–h1: T2WI images. e2–h2: T2WI ROIs. e3–h3: Predictions 2. e4–h4: Overlaps 2. **c** i1–l1: CE-T1WI images. i2–l2: CE-T1WI ROIs. i3–l3: Predictions 3. i4–l4: Overlaps 3. CE-T1WI, Contrast-enhanced T1-weighted imaging; DWI, Diffusion-weighted imaging; ROI, Region of interest; T2WI, T2-weighted imaging

As shown in Table 4, the quantitative evaluation metrics of automated segmentation are calculated based on the image level of the three MRI sequences. During the validation phase, the average values of DSC and Jaccard on DWI, T2WI, and CE-T1WI images were (85%, 74%), (78%, 64%), and (72%, 58%), respectively. Figure 4 demonstrates four groups of segmentation samples from different sequences in the test cohort. The mean DSC and Jaccard values were 83% (81−85%) and 71% (69–74%), 79% (75–82%) and 65% (61–69%), 74% (71–76%) and 59% (56–62%), respectively for DWI, T2WI, and CE-T1WI. The validation and testing results (Hypothesis i-b) both suggested that the segmentation performance on DWI images was better than the other two in the assessment of DSC and Jaccard values, which was similar to the finding in the detection performance.

**Table 4:** The test performance for segmenting multi-parametric MRI images (Center 1)

	Training and validation set			Internal testing set		
Modality	Slice	DSC	Jaccard	Slice	DSC	Jaccard
DWI (*b* = 1,000)	347	87%	77%	112	83% (81–85%)	71% (69–74%)
	115	85%	74%			
T2WI	332	84%	72%	111	79% (75–82%)	65% (61–69%)
	118	78%	64%			
CE-TIWI	694	86%	76%	294	74% (71–76%)	59% (56–62%)
	322	72%	58%			
Mixed (200)	1,373	65%	50%	517	54% (48–61%)	41% (36–47%)
	555	56%	43%			
Mixed (500)	1,373	64%	48%	517	57% (50–64%)	44% (38–50%)
	555	58%	45%			

*CE-T1WI* Contrast-enhanced T1-weighted imaging, *DSC* Dice similarity coefficient, *DWI* Diffusion-weighted imaging, *MRI* Magnetic resonance imaging, *T2WI* T2-weighted imaging

Mixed experiments were conducted by inputting images from the three sequences into one single DoubleU-Net model, as the last four rows of Table 4. The test results with DSCs of 54% (48–61%, 200 epochs) and 57% (50–64%, 500 epochs) prove that processing the images through three separate networks can achieve more desired outcomes. These results indicate the rationale for designing three independent DoubleU-Nets in the segmentation phase, as the domain differences between the various modalities have a lower impact on the learning of each channel.

To evaluate the clinical utility of this model, we performed a hypothesis test comparing the time taken by the pipeline with the time required by a GR. The time costs of the pipeline and the GR in actual clinical practice are summarized in Table 5. Considering all 20 cases from the testing set as the benchmark, the human expert spent average times of 50.05 s, 71.40 s, and 128.40 s (total time: 249.85 s) delineating the DWI, T2WI, CE-T1WI images, respectively, from one case. In contrast, the mean time to process one case by the DL pipeline was 10.07 s, 9.81 s, and 31.36 s (total time: 51.24), respectively. The results highlight a substantial time reduction of the pipeline, which was close to four-fifths of the total time under visual observation. These findings suggested the effectiveness of the end-to-end approach in promoting the clinical efficiency of cervical cancer, especially for the processes of image reading and interpreting.

**Table 5:** Time comparison between GR and the DL pipeline

	DWI		T2WI		CE-TIWI	
ID	GR time (s)	DL time (s)	GR time (s)	DL time (s)	GR time (s)	DL time (s)
1	58	9.45	64	9.62	160	35.92
2	32	9.28	40	9.39	59	29.67
3	24	9.28	60	9.39	71	26.88
4	31	10.85	71	9.39	75	26.73
5	62	10.21	90	11.14	103	35.87
6	67	10.97	115	9.69	164	27.27
7	94	11.09	112	9.84	280	27.46
8	28	9.33	52	9.47	67	26.78
9	112	10.57	124	10.83	328	36.94
10	64	11.03	80	9.69	222	27.12
11	83	11.15	97	9.84	216	27.66
12	38	11.03	77	9.69	163	27.42
13	32	10.09	56	10.23	68	35.68
14	80	9.63	82	9.76	167	36.41
15	34	10.15	36	10.23	76	35.68
16	20	8.52	44	8.63	46	29.67
17	13	9.16	45	9.24	33	35.34
18	48	10.15	52	10.30	70	29.96
19	42	9.39	83	9.54	102	35.82
20	39	10.15	48	10.30	98	32.94
Mean	50.05	10.07	71.40	9.81	128.40	31.36
HT	*T* = 6.83, *p* < 0.001^*^		*T* = 10.58, *p* < 0.001^*^		*Z* = -3.88, *p* < 0.001^#^	

*CE-T1WI* Contrast-enhanced T1-weighted imaging, *DL* Deep learning, *DWI* Diffusion-weighted imaging, *GR* Gynecologic radiologist, *HT* Hypothesis testing, *T2WI* T2-weighted imaging

^*^ Paired *t*-test

^#^ Wilcoxon signed-rank test

The *T*-scores (DWI, GR *versus* DL pipeline, *T* = 6.83, *p* < 0.001; T2WI, GR *versus* DL pipeline, *T* = 10.58, *p* < 0.001) confirmed that the differences were statistically significant, and the *Z*-score (CE-T1WI, GR *versus* DL pipeline, *Z* = -3.88, *p* < 0.001) also proved that the difference had existed in the distribution. These results further support the credibility of the outcomes that the pipeline realized (Hypothesis ii).

The patient-level generalization performance (Hypothesis iii) in the external testing set (Center 2) is presented in Table 6. The data parameters of Center 2 were distinct from those of Center 1, with a DWI b-value of 800. The resolution and spacing of the original images were also different. Straightforwardly, the trained pipeline in Center 1 was tested in Center 2 with no fine-tuning process, achieving the DSCs of 60% (53–67%), 72% (68–77%), and 70% (62–78%) on a per-patient basis. The pipeline maintained the DSC values greater than 70% for both T2 and DCE images, and the finding also demonstrated a certain level of robustness in DWI images, even with significant differences in *b*-values.

**Table 6:** The segmentation performance in cross-center validation (Center 2)

	External testing set			
Modality	Patient	Slice	DSC	Jaccard
DWI (*b* = 800)	25	122	60% (53–67%)	45% (37–54%)
T2WI	25	121	72% (68–77%)	57% (51–63%)
CE-T1WI	25	215	70% (62–78%)	57% (48–66%)

*CE-T1WI* Contrast-enhanced T1-weighted imaging, *DSC* Dice similarity coefficient, *DWI* Diffusion-weighted imaging, *T2WI* T2-weighted imaging

## Discussion

Accurate detection and segmentation of cervical cancer lesions have a vital role in the diagnosis, treatment planning, and evaluation of this prevalent malignancy. In recent years, multi-sequence segmentation has proven significant potential in cervical cancer applications [37, 38]. However, the manual recognition and segmentation of cervical cancer require high labor intensity due to the diversity in morphology and location of cervical cancer. Also, the integration of cervical cancer radiomics with clinical information, genomics, and histopathological data is required.

In this paper, we proposed a two-stage pipeline targeting automated detection and segmentation for cervical cancer on three different MRI sequences (DWI, T2WI, CE-T1WI) based on the advantages of ConvNeXt in classification and DoubleU-Net in segmentation. The external testing set results indicated that the end-to-end method could increase competitive recognition and segmentation accuracy with less manual intervention. The DL-based recognition network achieved overall results of 93% (92–94%), 93% (92–94%), 93% (92–94%), 0.90 (0.89–0.91), and 0.93 (0.92–0.94) for accuracy, precision, recall, κ, and F1-score, respectively. As for the next segmentation performance, the mean values of DSC on DWI, T2WI, and CE-T1WI images yielded 83% (81–85%), 79% (75–82%), and 74% (71–76%), respectively. When generalized to cross-center validation, the pipeline achieved promising and robust results in external validation at another center without any fine-tuning or retraining. Considering the time comparison of each sequence with the human gynecologist, the testing results demonstrated the advancement of the developed pipeline.

Our findings are consistent with prior research. In addition, we presented some valuable new data. Previous studies have mainly focused on the traditional voxel classification method (non-DL-based method) and DL-based algorithms with single-sequence MRI or CT images, and there has been relatively limited systematic research on the DL-based segmentation performance across different MRI sequences. Torheim et al [30] utilized voxel classification grounded on Fisher’s Linear Discriminant Analysis for multi-parametric MRI segmentation consisting of T2WI, T1WI, and dynamic contrast-enhanced (DCE) sequences, finding DSC values between 0.18 and 0.20 for the T2WI and T1WI images. Even when including DCE-MRI features, the models had merely higher values of DSC (0.41–0.44). In contrast, our results demonstrated the advantages of segmentation accuracy by using a DL-based pipeline for multi-parametric MRI segmentation. Moreover, Rigaud et al [39] developed 2D DeepLab v3+ to segment CT images for optimizing radiation dose and reducing toxicities in cervical cancer radiotherapy research. The average DSC values of the 2D model achieved 0.81. Despite the different imaging modalities of CT and MRI, these results align with our results in an approximate numerical range of DSC values. In addition, Kano et al [22] created an automatic segmentation model for 98 cervical cancer patients (DWI images) using a combination of 2D UNet and 3D UNet. The DSC ranged from 0.13 to 0.93 (median 0.83, mean 0.77). Our pipeline was also consistent with the DSC range exclusively on the DWI sequence and attained an advanced mean DSC of 0.83. Hodneland et al [21] applied an enhanced residual UNet model for three-dimensional segmentation of primary cervical cancer lesions in pelvic MRI (T2 images). The DL model achieved median DSC values of 0.60 and 0.58 compared to two human radiologists. According to practical experience, purely 3D models often decrease segmentation accuracy due to the narrow and slender structure of the cervix. This also explains the rationale for the technological choice, mainly using 2D slices and 2D models in our study.

Considering the clinical practice, the developed pipeline verified the effectiveness of less time consumption compared with the GR. The ratio of time-saving was 80%, 86%, and 76% on DWI, T2WI, and CE-T1WI images, respectively. These findings imply that the end-to-end Cer-ConvN3Unet pipeline not only improves the accuracy of lesion identification but also holds great promise for accelerating treatment-related processes such as tumor measurement and radiotherapy target delineation [27, 40–42]. This fully automated lesion identification and segmentation method is beneficial to freeing radiologists from labor-intensive contouring tasks, but more importantly, it provides oncologists with a promising auxiliary tool to treatment plans, thus facilitating the clinical efficacy of cervical cancer.

Contrast analysis of the experimental results using this pipeline among different MRI sequences provides more relevant information. In the context of automatic recognition tasks, the DL model demonstrates varying performance across different sequences, with rankings from highest to lowest as DWI, T2WI, and CE-T1WI. The performance ranking in the segmentation task is the same as that of the detection. The main reason identified after analysis is that T2WI and CE-T1WI images contain more spatial and structural information, leading to increased interference from surrounding tissues. In contrast, DWI images exhibit high-intensity signals with less interference from surrounding information. Hence, the experimental results are consistent with the radiological characteristics of the three sequences.

The present study has some limitations. First, the 2D MRI slices primarily focused on the axial plane. Further validation of the pipeline performance could be conducted using sagittal and coronal plane slices. Second, our current pipeline was implemented on 2D CNNs. A fully 3D pipeline may provide enhanced convenience in practical applications. The subsequent challenges of developing and training 3D networks with the guarantee of segmentation accuracy also require further exploration and consideration. Third, incorporating more scanners from additional vendors is adequate to reduce the equipment-based sensitivity of the detection and segmentation models. Finally, the experiments relied on a multi-center retrospective case series, and the clinical utility of the pipeline should be broadly examined in large-sample and multi-center prospective cohorts. In the future, the pipeline will have a high potential for automated segmentation of multi-sequence target volumes and organs at risk during radiotherapy [43, 44]. Meantime, the study will hopefully assist multi-parametric MRI-based radiomics analysis and help construct a DL-based multi-parametric radiomics system for cervical cancer prognosis/prediction [42, 45]. Further work could explore including MRI images, genetic data, and histopathological data to create a more holistic model, allowing for personalized and precise treatment strategies [46, 47].

In conclusion, the proposed two-stage multi-parametric pipeline of cervical cancer, *i.e.*, Cer-ConvN3Unet, holds significant promise for improving diagnosis, treatment planning, and personalized medicine approaches. This end-to-end method enhances the accuracy of lesion identification, assists in treatment optimization, and provides valuable quantitative information for treatment evaluation. The pipeline shows great potential and may become a very useful alternative tool for gynecologists and radiologists in the routine reading and processing of cervical cancer MRI images. The pipeline may be used as a foundation for downstream lesion-related research, significantly decreasing labor-intensive delineation tasks.

## Supplementary information

**Additional file 1**: **Fig. S1.** Training curves of the multi-parametric MRI slices detection module during training, using different pre-trained ConvNeXt variants and ImageNet datasets (ImageNet-1K, ImageNet-22K). **(a)** ConvNeXt-T, ImageNet-1K. **(b)** ConvNeXt-S, ImageNet-1K. **(c)** ConvNeXt-B, ImageNet-1K. **(d)** ConvNeXt-B, ImageNet-22K. **(e)** ConvNeXt-L, ImageNet-1K. **(f)** ConvNeXt-L, ImageNet-22K. **(g)** ConvNeXt-XL, ImageNet-22K. **Fig. S2.** Training curves of the independent three-channel segmentation module on different MRI sequences, and loss curves of two mixed experiments. **(a)** DWI images. **(b)** T2WI images. **(c)** CE-T1WI images. **(d)** Mixed experiment (200 epochs). **(e)** Mixed experiment (500 epochs).

## Data Availability

The datasets are available from the first author upon reasonable request.

## Abbreviations

AUC: Area under the curve
CE-T1WI: Contrast-enhanced T1-weighted imaging
CT: Computed tomography
DSC: Dice similarity coefficient
DWI: Diffusion-weighted imaging
GR: Gynecologic radiologist
MRI: Magnetic resonance imaging
ROC: Receiver operating characteristic
ROI: Region of interest
T2WI: T2-weighted imaging
