# Supplementary material for: Cer-ConvN3Unet: an end-to-end multi-parametric MRI-based pipeline for automated detection and segmentation of cervical cancer
Source: Eur Radiol Exp. 2025 Feb 18;9:20. doi: 10.1186/s41747-025-00557-2 (PMC11836247; doi:10.1186/s41747-025-00557-2)
Supplement: Supplementary file 1 — Additional file 1: Fig. S1. Training curves of the multi-parametric MRI slices detection module during training, using different pre-trained ConvNeXt variants and ImageNet datasets (ImageNet-1K, ImageNet-22K). (a) ConvNeXt-T, ImageNet-1K. (b) ConvNeXt-S, ImageNet-1K. (c) ConvNeXt-B, ImageNet-1K. (d) ConvNeXt-B, ImageNet-22K. (e) ConvNeXt-L, ImageNet-1K. (f) ConvNeXt-L, ImageNet-22K. (g) ConvNeXt-XL, ImageNet-22K. Fig. S2. Training curves of the independent three-channel segmentation module on different MRI sequences, and loss curves of two mixed experiments. (a) DWI images. (b) T2WI images. (c) CE-T1WI images. (d) Mixed experiment (200 epochs). (e) Mixed experiment (500 epochs). [file 41747_2025_557_MOESM1_ESM.pdf]

Cer-ConvN3Unet: an end-to-end multi-parametric MRI-based pipeline for automated detection and segmentation of cervical cancer

ELECTRONIC SUPPLEMENTARY MATERIAL

Eur Radiol Exp (2025) Xia SJ, Zhao B, Li YM, et al.

Supplementary Figure 1:

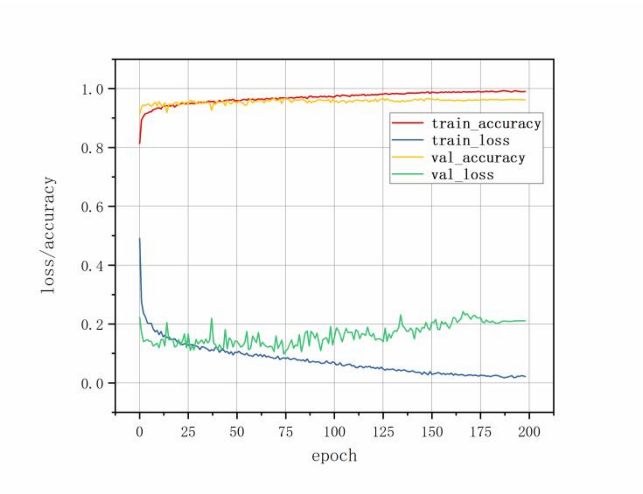

(a)

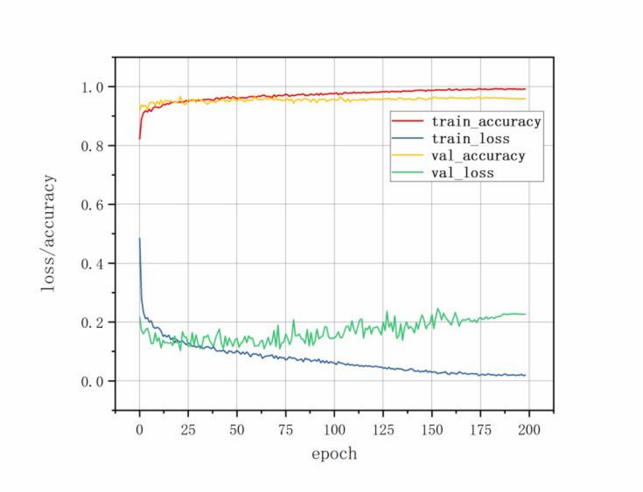

(b)

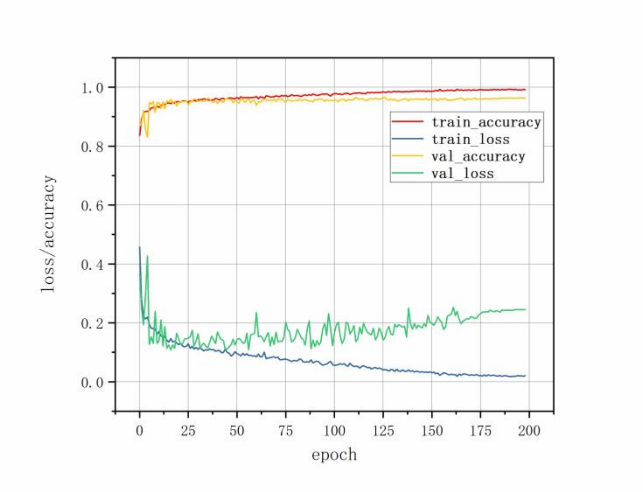

(c)

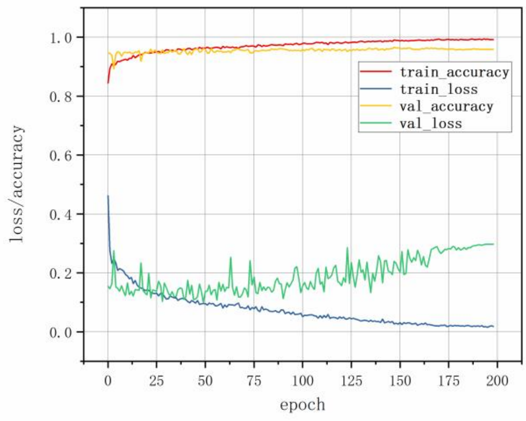

1.0
0.8
0.6
0.4
0.2
0.0
loss/accuracy
0
25
50
75
100
125
150
175
200
epoch
train_accuracy
train_loss
val_accuracy
val_loss

(d)

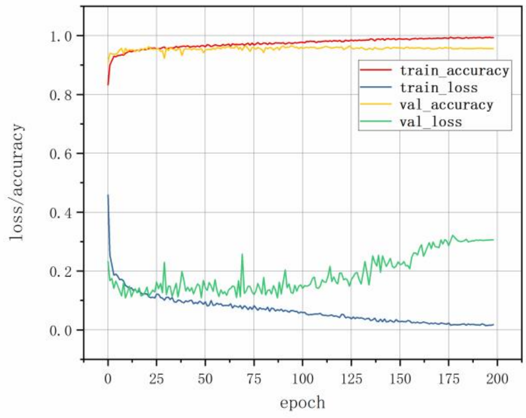

1.0
0.8
0.6
0.4
0.2
0.0
loss/accuracy
0
25
50
75
100
125
150
175
200
epoch
train_accuracy
train_loss
val_accuracy
val_loss

(e)

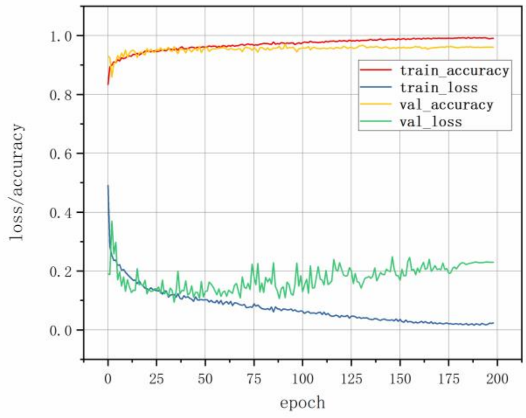

1.0
0.8
0.6
0.4
0.2
0.0
loss/accuracy
0
25
50
75
100
125
150
175
200
epoch
train_accuracy
train_loss
val_accuracy
val_loss

(f)

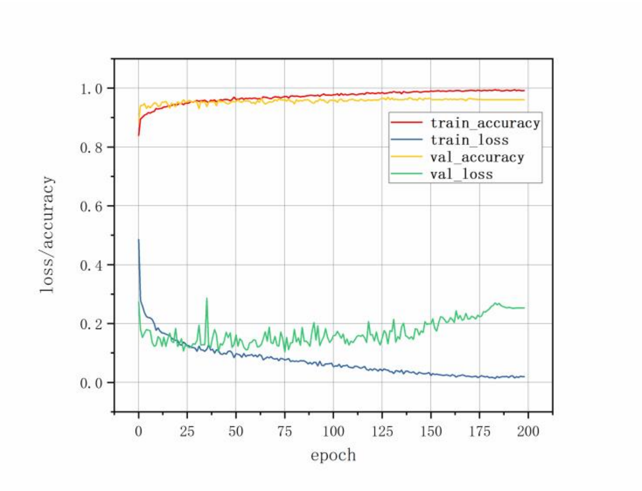

(g)

Fig. S1 Training curves of the multi-parametric MRI slices detection module during training, using different pre-trained ConvNeXt variants and ImageNet datasets (ImageNet-1K, ImageNet-22K). **(a)** ConvNeXt-T, ImageNet-1K. **(b)** ConvNeXt-S, ImageNet-1K. **(c)** ConvNeXt-B, ImageNet-1K. **(d)** ConvNeXt-B, ImageNet-22K. **(e)** ConvNeXt-L, ImageNet-1K. **(f)** ConvNeXt-L, ImageNet-22K. **(g)** ConvNeXt-XL, ImageNet-22K.

Supplementary Figure 2:

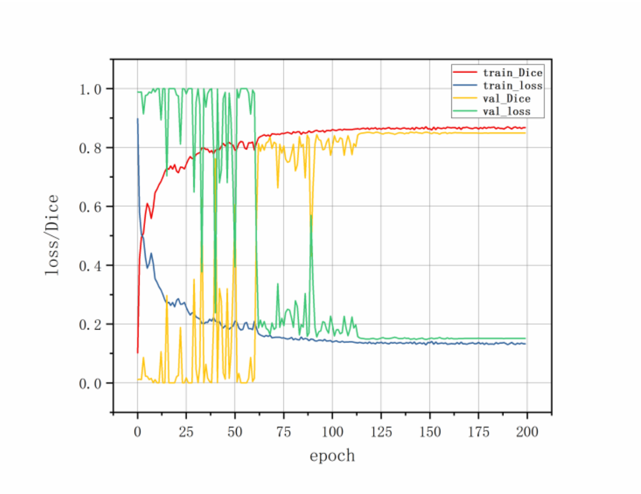

(a)

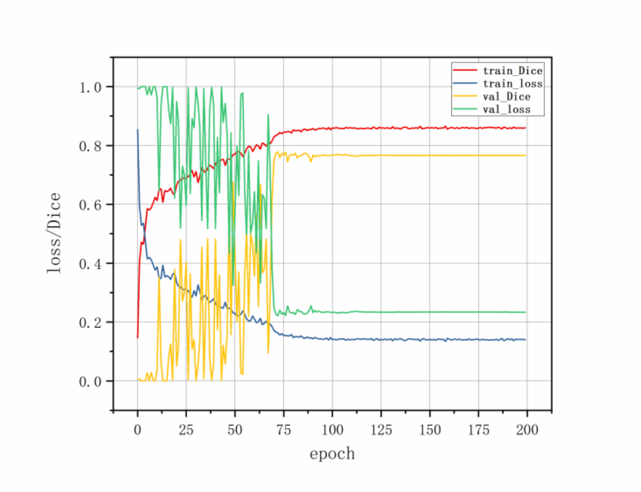

(b)

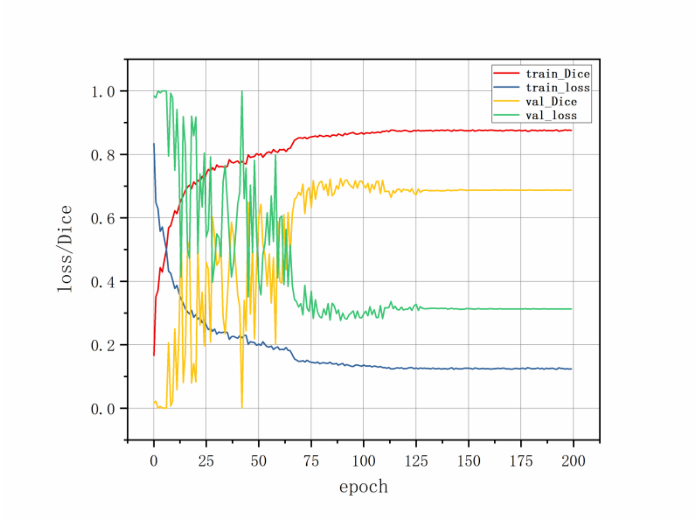

(c)

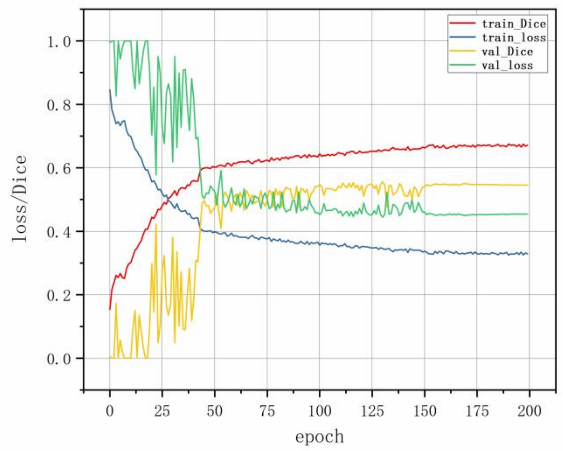

(d)

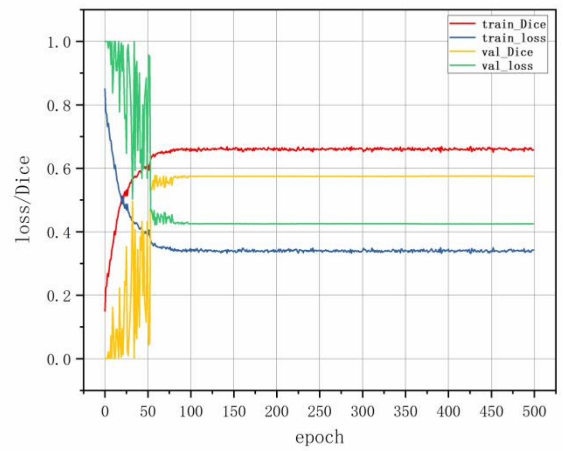

(e)

Fig. S2 Training curves of the independent three-channel segmentation module on different MRI sequences, and loss curves of two mixed experiments. **(a)** DWI images. **(b)** T2WI images. **(c)** CE-T1WI images. **(d)** Mixed experiment (200 epochs). **(e)** Mixed experiment (500 epochs).
